# MRI findings of malignant transformation arising from mature cystic teratoma of the ovary: comparison with benign mature cystic teratoma

**DOI:** 10.1007/s11604-023-01521-z

**Published:** 2023-12-26

**Authors:** Masaya Kawaguchi, Hiroki Kato, Tatsuro Furui, Yoshifumi Noda, Fuminori Hyodo, Tatsuhiko Miyazaki, Masayuki Matsuo

**Affiliations:** 1 Department of Radiology, Gifu University, 1-1 Yanagido, Gifu, 501-1194 Japan; 2 Department of Radiology, Ogaki Municipal Hospital, 4-86 Minaminokawacho, Ogaki, 503-0864 Japan; 3 Department of Obstetrics and Gynecology, Gifu University, Gifu, Japan; 4 Center for One Medicine Innovative Translational Research (COMIT), Institute for Advanced Study, Gifu University, Gifu, Japan; 5 Department of Pathology, Gifu University, Gifu, Japan

**Keywords:** Ovary, Teratoma, Malignant, Benign, MRI

## Abstract

**Objective:**

This study aimed to evaluate the efficacy of MRI findings to differentiate malignant transformation arising from mature cystic teratoma (MT-MCT) of the ovary from benign mature cystic teratoma (BMCT).

**Materials and methods:**

This study included 11 patients with histopathologically proven MT-MCT and 50 with BMCT. Overall, 7 patients with MT-MCT and all 50 with BMCT underwent unenhanced and contrast-enhanced MRIs and 4 with MT-MCT only underwent unenhanced MRIs. The MRI findings were evaluated and compared between the two diseases.

**Results:**

The median age (55 vs. 38 years, *p* < 0.01) and maximum diameter (109 vs. 65 mm, *p* < 0.01) were higher in MT-MCT than in BMCT. Fat component occupancy was lower in MT-MCT than in BMCT (median, 5% vs. 63%, *p* < 0.01). Only MT-MCT exhibited irregular tumor margins (64%), peritoneal dissemination (18%), and abnormal ascites (27%). The solid components were more commonly observed in MT-MCT than in BMCT (100% vs. 32%,* p* < 0.01) on contrast-enhanced images. The maximum diameter of solid components in MT-MCT was larger than that in BMCT (median, 61 mm vs. 14 mm, *p* < 0.01). In MT-MCT, the common configuration of solid components was endophytic or exophytic sessile (85%), whereas in BMCT, it was endophytic papillary (88%).

**Conclusion:**

Compared with BMCT, MT-MCT demonstrated a larger maximum diameter, lower occupancy rate of fat components, and sessile solid components. The characteristic configuration of solid components was endophytic or exophytic sessile in MT-MCT and endophytic papillary in BMCT.

## Introduction

Mature cystic teratomas (MCTs) of the ovary are the most common type of germ cell tumor accounting for 20% of all ovarian neoplasms. MCTs can occur in women of various ages, with the highest occurrence observed in women of reproductive age [1–3]. MCT is a tumor entirely formed of mature tissues derived from two or three germ layers: ectodermal, mesodermal, and endodermal [2].

When MCT components develop into somatic malignant tumors, they can change into malignant germ cell tumors, a condition known as malignant transformation arising from MCT (MT-MCT). The incidence of MT-MCT of the ovary is reported to be 0.2–0.8% [4–6]. Patients with MT-MCT of the ovary have a median age of 50–55 year [4–9]. Squamous cell carcinoma is the major histological subtype of malignant transformations, followed by adenocarcinoma and sarcoma [3, 10]. The typical therapies for MT-MCT of the ovary are the same as for ovarian carcinoma, including surgical removal and chemotherapy, whereas benign MCTs (BMCTs) are commonly treated with simple cystectomy or oophorectomy [3]. Recurrence rates following resection are 25–50% [4, 5] and 2% [11] in MT-MCT and BMCT of the ovary, respectively. MT-MCT of the ovary has a 5-year overall survival rate of 15–52% for all stages combined and 75.7% for stage I tumors [10], whereas BMCTs are not lethal unless they become MT-MCT. Because of the differences in prognosis and treatment strategies, proper distinction between MT-MCT and BMCT is critical.

According to the MRI findings of MT-MCT of the ovary, it is a huge mass with solid components that widely invades the nearby organs [8, 12–14]. Although there is a comparison study of CT imaging features between MT-MCT and BMCT of the ovary [15], to the best of our knowledge, no study has investigated the MRI differences between MT-MCT and BMCT of the ovary. Therefore, this study aimed to compare the MRI findings of MT-MCT and BMCT of the ovary.

## Methods

### Patients

The human research committee of our hospital’s institutional review board approved this study. This study was performed according to the Health Insurance Portability and Accountability Act of 1996. Because this is a retrospective study, informed consent was not required. From January 2008 to April 2023, patients with histopathologically confirmed MT-MCT of the ovary who underwent surgical removal at two Japanese institutions were searched and 11 patients with MT-MCT were identified. During the same span, we found 650 patients with histopathologically established BMCT of the ovary at a single Japanese hospital. Among them, we randomly selected 50 patients with BMCT who underwent preoperative contrast-enhanced MRI because we wanted to extract the same number of BMCT cases with solid components as MT-MCT. In a previous MRI study [16], 18.8% of BMCT cases had enhancing solid components. If 50 BMCTs are investigated, approximately 10 BMCT cases are supposed to have enhancing solid components. Consequently, this study included 11 patients with MT-MCT (age range, 28–83 year; mean age, 57 year) and 50 patients with BMCT (age range, 14–85 year; mean age, 41 year). Of the 11 patients, 10 with MT-MCT had squamous cell carcinoma, and one patient had poorly differentiated thyroid carcinoma. The clinical stages of MT-MCT based on revised 2018 International Federation of Gynecology and Obstetrics staging system were stage I (*n* = 5), II (*n* = 1), III (*n* = 2), and IV (*n* = 3).

### MRI protocols

MRI was performed using a 1.5 T unit (Intera Achieva 1.5 T Pulsar; Philips Healthcare, Best, The Netherlands), a 1.5 T unit (SIGNA Explorer; GE Medical Systems, The United States), or a 3.0 T unit (Intera Achieva 3.0 T Quasar Dual; Philips Healthcare, Best, The Netherlands). Seven patients with MT-MCT and all 50 with BMCT unenhanced underwent contrast-enhanced MRI, whereas 4 with MT-MCT underwent unenhanced MRI alone. All MRI images were obtained at a section thickness of 4–8 mm with 1–2 mm intersection gap and a 26 × 26–36 × 36-cm field of view. T2-weighted fast spin-echo (TR/TE, 2000–7000/81–100 ms), T1-weighted spin-echo (TR/TE, 566–728/6.2–17 ms), and fat-suppressed T1-weighted fast spin-echo (TR/TE, 650–816/6.7–15 ms) were obtained in axial and coronal or sagittal planes. In 8 patients with MT-MCT and 50 with BMCT, diffusion-weighted single shot spin-echo echo-planar (TR/TE, 2394–5000/69–72 ms; b-value = 0 and 1000 s/mm^2^) images were obtained in axial planes. In 7 patients with MT-MCT and 50 with BMCT, axial and coronal or sagittal fat-suppressed gadolinium-enhanced T1-weighted spin-echo (TR/TE, 650–816/6.7–17 ms) images were obtained after the intravenous injection of 0.1 mmol/kg of gadopentetate dimeglumine (Magnevist, Bayer HealthCare, Leverkusen, Germany) or gadobutrol (Gadavist, Bayer HealthCare, Leverkusen, Germany).

### Imaging analysis

All images were assessed independently by two radiologists with 23- and 9-year post-training experience in gynecological imaging, and any disputes were resolved via consensus. The reviewers were blinded to the clinical information and pathological diagnosis.

First, the maximum diameter of the lesion was assessed quantitatively. Laterality (unilateral or bilateral), lobulation (unilocular or multilocular), fat component presence, irregular tumor margins, lymphadenopathy, peritoneal dissemination, and abnormal ascites were also evaluated. Fat components were analyzed on T1- and fat-suppressed T1-weighted images, and the occupancy rate of fat components was evaluated if fat components existed. For all images on which teratomas were visible, areas of the whole tumor and fat components were separately measured by tracing the contour manually. The occupancy rate of fat components was calculated as the fat component-to-whole tumor ratios [17]. Irregular tumor margins were defined as uneven or rough, not smooth. Lymph nodes were considered to have lymphadenopathy when a node is more than 8 mm in short-axis diameter in the pelvis [18]. Peritoneal dissemination was defined as nodular or smooth thickening of peritoneum. Abnormal ascites was defined as the presence of ascites exceeding the level of uterine fundus and/or filling the pelvic cavity, whereas physiological ascites was defined as fluid at the Douglas level [19].

**Fig. 1:**
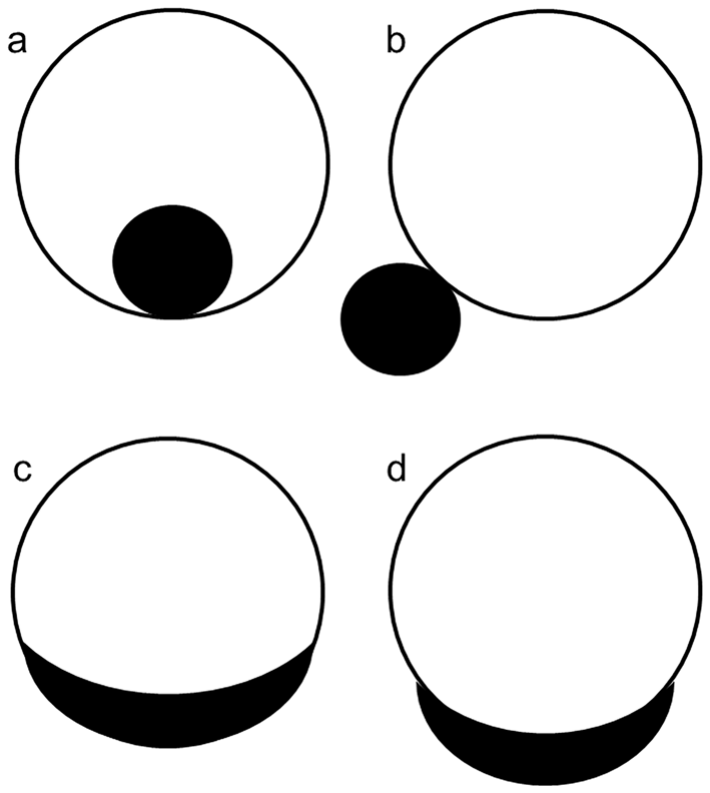
Schematic diagram of configuration of solid components. The configuration of solid components was classified into four categories; (**a**) endophytic papillary, (**b**) exophytic papillary, (**c**) endophytic sessile, and (**d**) exophytic sessile

Second, contrast-enhanced images were used to evaluate solid components. Enhanced nodule or thickened septa (> 5 mm) were considered solid components. If the solid components were observed, the maximum diameter of the solid components was quantitatively measured. Subsequently, the number (single or multiple), configuration (endophytic papillary, exophytic papillary, exophytic sessile, or endophytic sessile), presence of internal fat, and necrosis were determined on contrast-enhanced images. Fat signal intensity on the T1- and fat-suppressed T1-weighted images within the enhanced areas was used to characterize internal fat presence. Configuration was defined as follows: (a) endophytic papillary; papillary growth inside the cyst wall with an acute angle, (b) exophytic papillary; papillary growth outside the cyst wall with acute angle (c) endophytic sessile; broad-based growth inside the cyst wall with an obtuse angle, and (d) exophytic sessile; broad-based growth outside the cyst wall with an obtuse angle (Fig. 1).

Finally, the signal intensity ratio on T1-, T2-, and contrast-enhanced T1-weighted images, and apparent diffusion coefficient (ADC) value was evaluated. A reviewer defined the regions of interest (ROI) in the solid components and iliopsoas muscle on the T1-, T2-, and contrast-enhanced T1-weighted images, and recorded these signal intensities. The ratio of solid components to muscle signal intensity was calculated. Solid components ADC values were also measured on the ADC maps by placing ROI over the solid components. By referring to T2- and contrast-enhanced T1-weighted images, ROIs on the ADC maps were placed as broadly as possible within the solid components while omitting necrotic or cystic areas in solid components.

### Statistical analysis

All statistical analyses were performed with EZR (Saitama Medical Center, Jichi Medical University, Saitama, Japan), which is a graphical user interface for R (The R Foundation for Statistical Computing, Vienna, Austria). More precisely, it is a modified version of R commander designed to add statistical functions frequently used in biostatistics [20]. Mann–Whitney *U* test was used to compare the quantitative results between MT-MCT and BMCT. The Fisher exact test was performed to compare the qualitative results between MT-MCT and BMCT. Receiver operating characteristics curve analysis was used to determine the performance of the occupancy rate of fat components and area under the curve (AUC) was calculated to establish the optimal cut-off value for differentiating MT-MCT from BMCT. *p* values < 0.05 were considered significant. Inter-observer variability of qualitative assessments was assessed using kappa statistics.

## Results

**Fig. 2:**
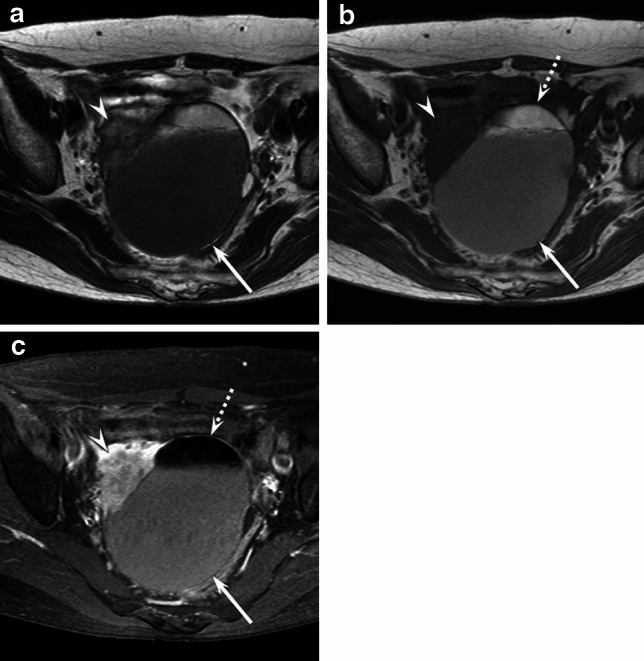
A 70-year-old woman with MT-MCT (stage IC1). **a**. Axial T2-weighted image showing a fat-containing mass (arrow) with exophytic sessile solid components and irregular margin (arrowhead). **b**. Axial T1-weighted image showing a fat-containing mass (arrow) with exophytic sessile solid components (arrowhead). The occupancy rate of fat components accounting for 6% (dotted arrow). **c**. Axial fat-suppressed contrast-enhanced T1-weighted image showing a fat-containing mass (arrow) with fat-suppressed area (dotted arrow) and exophytic sessile solid components (arrowhead)

**Fig. 3:**
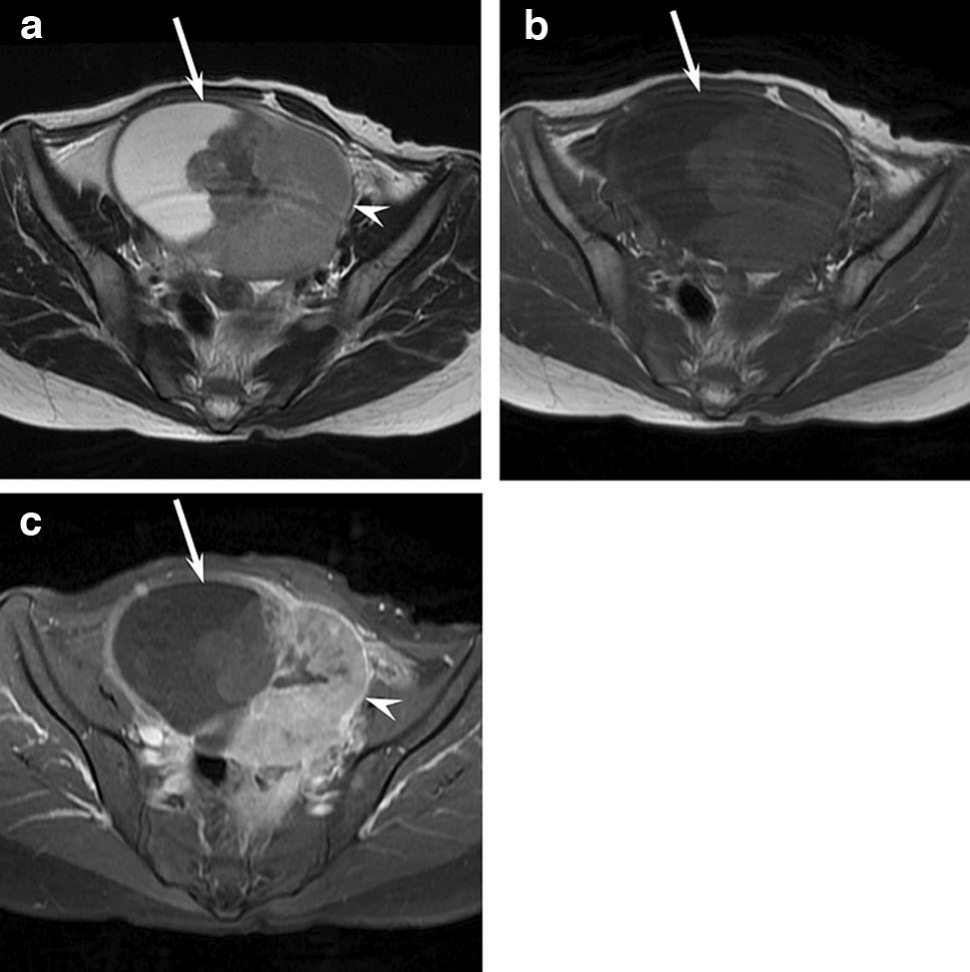
A 49-year-old woman with MT-MCT (stage IIA). **a**. Axial T2-weighted image showing a cystic mass (arrow) with endophytic sessile solid components (arrowhead). **b**. Axial T1-weighted image showing a cystic mass (arrow) without fatty components. **c**. Axial fat-suppressed contrast-enhanced T1-weighted image showing a cystic mass (arrow) with endophytic sessile solid components (arrowhead)

**Fig. 4:**
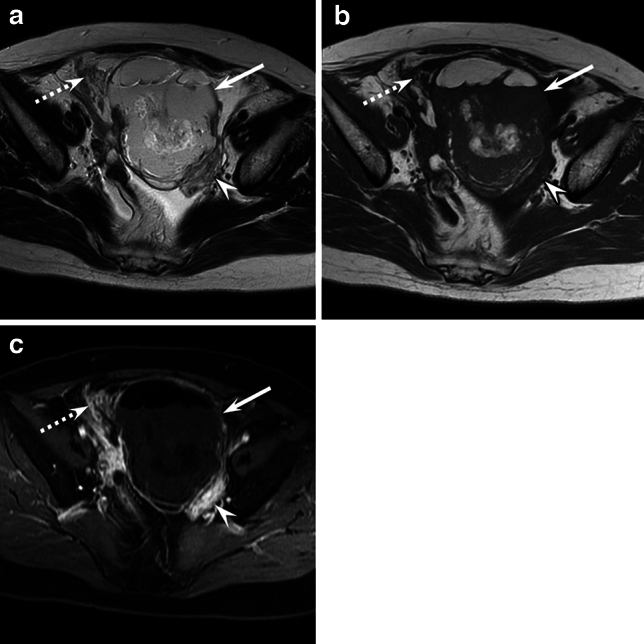
A 55-year-old woman with MT-MCT (stage IIIC). Axial T2-weighted image (**a**), T1-weighted image (**b**), and axial fat-suppressed contrast-enhanced T1-weighted image (**c**) showing an ill-defined fat-containing mass (arrow) with exophytic sessile solid components (arrowhead) and peritoneal dissemination (dotted arrow). The occupancy rate of fat components accounting for 23%

**Fig. 5:**
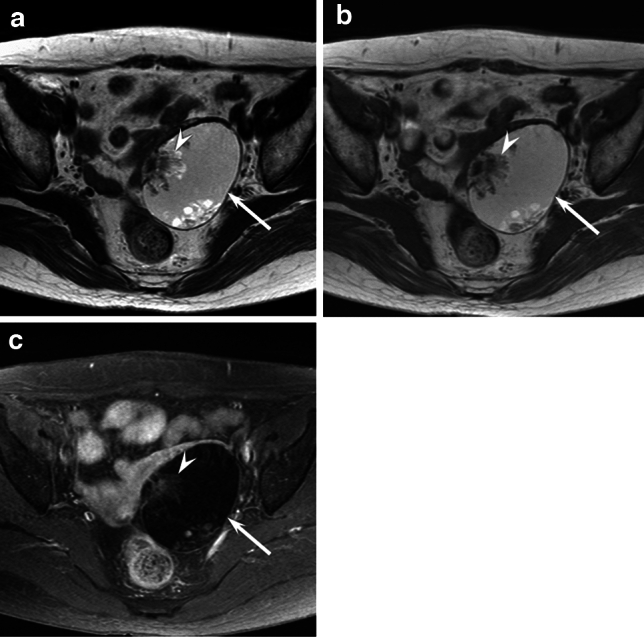
A 66-year-old woman with BMCT. Axial T2-weighted image (**a**), T1-weighted image (**b**), and axial fat-suppressed contrast-enhanced T1-weighted image (**c**) showing a well-defined fat-containing mass (arrow) with endophytic papillary solid components (arrowhead). The occupancy rate of fat components accounting for 93%

Clinical and imaging findings are summarized in Table 1. The median age (55 vs. 38 years, *p* < 0.01) and the maximum diameter (109 vs. 65 mm, *p* < 0.01) were higher in the patients with MT-MCT than in the patients with BMCT. Although there was no significant difference in the presence of fat components between MT-MCT and BMCT (*p* = 0.55), the occupancy rate of fat components was lower in patients with MT-MCT than in patients with BMCT (median, 5% vs. 63%, *p* < 0.01) (Figs. 2, 3, 4, 5). The AUC of the occupancy rate of fat components for predicting MT-MCT was 0.847. The sensitivity and specificity using a cut-off value of 23.8% were 1.00 and 0.78, respectively. Irregular tumor margin (7/11, 64%), peritoneal dissemination (2/11, 18%), and abnormal ascites (3/11, 27%) were observed only in MT-MCT; however, there was no significant difference in laterality (*p* > 0.99), lobulation (*p* = 0.50), or lymphadenopathy (*p* > 0.99).

**Table 1:** Clinical and imaging findings of MT-MCT and BMCT of the ovary

	MT-MCT (*n* = 11)	BMCT (*n* = 50)	*p* value	Kappa
Age (year)	55 [52–71]	38 [28–50]	< 0.01*	
Maximum diameter (mm)	109 [94–131]	65 [53–84]	< 0.01*	
Laterality–unilateral	10 (9)	42 (84)	> 0.99	
Lobulation–multilocular	6 (55)	33 (66)	0.50	0.40
Fat components	11 (100)	46 (92)	> 0.99	0.55
Occupancy rate of fat (%)	4.9 [1.8–12.9]	63.3 [28.6–98.8]	< 0.01*	
Irregular tumor margins	7 (64)	0 (0)	< 0.01*	1.00
Lymphadenopathy	0 (0)	0 (0)	> 0.99	NA
Peritoneal dissemination	2 (18)	0 (0)	0.03*	0.66
Abnormal ascites	3 (27)	0 (0)	< 0.01*	1.00

Quantitative data are expressed as medians with interquartile in square brackets

Qualitative data are expressed as raw numbers with percentages in parentheses

*MT-MCT* malignant transformation arising from mature cystic teratoma, *BMCT* benign mature cystic teratoma, *NA* not applicable

^*^Significant difference was observed in MT-MCT and BMCT (*p* < 0.05)

The imaging findings of solid components on the contrast-enhanced image are summarized in Table 2. Solid components were more common in patients with MT-MCT than in patients with BMCT (100% vs. 32%,* p* < 0.01). The maximum diameter of solid components in patients with MT-MCT was larger than in patients with BMCT (median, 61 vs. 14 mm, *p* < 0.01). In MT-MCT, the common configuration of solid components was endophytic or exophytic sessile (85%), whereas in BMCT, it was endophytic papillary (88%, *p* < 0.01) (Figs. 2, 3, 4, 5). In MT-MCT, the signal intensity ratio of solid components on T2-weighted images was lower than in BMCT (median, 2.24 vs. 3.25, *p* = 0.01). There was no significant difference in the number (*p* = 0.52), internal fat (*p* = 0.53), necrosis (*p* = 0.19), signal intensity ratio on T1- and contrast-enhanced T1-weighted image (*p* = 0.09, 0.62, respectively), or ADC value (*p* = 0.97) of solid components.

**Table 2:** Imaging findings of solid components of MT-MCT and BMCT of the ovary on contrast-enhanced images

	MT-MCT (*n* = 7)	BMCT (*n* = 50)	*p* value	Kappa
Solid components	7 (100)	16 (32)	< 0.01*	0.56
	*n* = 7	*n* = 16		
Maximum diameter (mm)	61 [46–90]	14 [12–24]	< 0.01*	
Number–multiple	2 (29)	2 (12)	0.52	0.38
Configuration			< 0.01*	0.63
Endophytic papillary	0 (0)	14 (88)		
Exophytic papillary	1 (14)	1 (6)		
Endophytic sessile	2 (29)	1 (6)		
Exophytic sessile	4 (57)	0 (0)		
Internal fat	0 (0)	3 (19)	0.53	0.46
Necrosis	5 (71)	6 (38)	0.19	0.26
SIR on T1WI	0.92 [0.80–1.00]	1.03 [0.95–1.31]	0.09	
SIR on T2WI	2.24 [1.87–2.43]	3.25 [2.86–4.51]	0.01*	
SIR on contrast-enhanced T1WI	1.69 [1.42–2.17]	1.34 [1.21–1.88]	0.62	
ADC value	*n* = 6 1.20 [1.07–1.28]	*n* = 16 1.23 [0.93–1.61]	0.97	

Quantitative data are expressed as medians with interquartile in square brackets

Qualitative data are expressed as raw numbers with percentages in parentheses

*MT-MCT* malignant transformation arising from mature cystic teratoma, *BMCT* benign mature cystic teratoma, *SIR* signal intensity ratio, *T1WI* T1-weighted image, *T2WI* T2-weighted image, *ADC* apparent diffusion coefficient

^*^Significant difference was observed in MT-MCT and BMCT (*p* < 0.05)

The imaging findings of stage I–II (*n* = 6) and III–IV (*n* = 5) MT-MCT of the ovary are summarized in Table 3. Peritoneal dissemination (2/5, 40%) and abnormal ascites (3/5, 60%) were observed only in stage III–IV MT-MCT of the ovary. However, no significant difference in all imaging findings was observed between stage I–II and III–IV MT-MCT of the ovary.

**Table 3:** Imaging findings of stage I–II and III–IV MT-MCT of the ovary

	Stage I–II (*n* = 6)	Stage III–IV (*n* = 5)	*p* value
Maximum diameter (mm)	101 [79–125]	109 [101–134]	0.41
Laterality–unilateral	6 (100)	4 (80)	0.46
Lobulation–multilocular	4 (67)	5 (100)	> 0.99
Fat components	6 (100)	5 (100)	> 0.99
Occupancy rate of fat (%)	2.8 [1.7–7.0]	6.2 [4.8–17]	0.43
Irregular tumor margins	2 (33)	5 (100)	0.06
Lymphadenopathy	0 (0)	0 (0)	> 0.99
Peritoneal dissemination	0 (0)	2 (40)	0.18
Abnormal ascites	0 (0)	3 (60)	0.06
Contrast-enhanced image	*n* = 4	*n* = 3	
Solid components	4 (100)	3 (100)	> 0.99
Maximum diameter (mm)	46 [33–63]	77 [69–89]	0.41
Number–multiple	1 (25)	1 (33)	> 0.99
Configuration			> 0.99
Endophytic papillary	0 (0)	0 (0)	
Exophytic papillary	1 (25)	0 (0)	
Endophytic sessile	1 (25)	1 (33)	
Exophytic sessile	2 (50)	2 (67)	
Internal fat	0 (0)	0 (0)	> 0.99
Necrosis	3 (75)	2 (67)	> 0.99
SIR on T1WI	0.77 [0.74–0.89]	0.96 [0.92–1.07]	0.40
SIR on T2WI	2.30 [2.03–2.62]	2.19 [1.95–2.33]	0.70
SIR on contrast-enhanced T1WI	1.38 [1.09–1.89]	1.83 [1.69–2.06]	0.70
ADC value	*n* = 3 1.29 [1.22–1.32]	*n* = 3 1.04 [0.99–1.14]	0.21

Quantitative data are expressed as medians with interquartile in square brackets

Qualitative data are expressed as raw numbers with percentages in parentheses

*MT-MCT* malignant transformation arising from mature cystic teratoma, *BMCT* benign mature cystic teratoma, *SIR* signal intensity ratio, *T1WI* T1-weighted image, *T2WI* T2-weighted image, *ADC* apparent diffusion coefficient

The Kappa values for the two observers exhibited fair agreement regarding the number of solid components, lobulation, and necrosis (0.26–0.40) and moderate agreement regarding the presence of fat, peritoneal dissemination, configuration, and solid components presence (0.55–0.66). There was a perfect agreement between irregular tumor margins and abnormal ascites.

## Discussion

The presence of solid components and a larger maximum diameter of the lesion were features of MT-MCT of the ovary in this study. The occupancy rate of fat components in MT-MCT was lower than that in BMCT. Only MT-MCT exhibited irregular tumor margins, peritoneal dissemination, and abnormal ascites. On contrast-enhanced images, solid components were more common in MT-MCT than in BMCT. In MT-MCT, the common configuration of solid components was endophytic or exophytic sessile, whereas in BMCT, it was endophytic papillary.

Herein, the maximum diameter of MT-MCT was larger than that of BMCT of the ovary. The mean maximum diameter of MT-MCT of the ovary is 10–14.5 cm (range, 1–40 cm) [4–8, 12], whereas that of BMCT is 6.7–8.6 cm (range, 1–21 cm) [11, 17, 21]. The larger size of the mass of > 10 cm is one of the important findings that suggests MT-MCT of the ovary.

According to this study, the occupancy rate of fat components was lower in MT-MCT than in BMCT of the ovary. In the previous MT-MCT cases with MRI or CT imaging [8, 12–14, 22–25], in 8/11 (73%) cases of MT-MCT, the occupancy rates of visible fat components on the images were < 50% in 8 of 11 (73%) patients. The decreased occupancy rate of fat components in MT-MCT can be attributed to the fact that increasing levels of malignant solid components reduce the relative portion of adipose tissue or that destroying sebaceous glands reduce sebaceous secretion. Two previous MT-MCT cases showed decreasing occupancy rate of fat components over the course of the disease [8]. Furthermore, the fat component occupancy in BMCT is 64.7% [17]. Thus, the lower rate of fat component is characteristic of MT-MCT of the ovary.

The present study found irregular margins, peritoneal dissemination, and abnormal ascites exclusives in MT-MCT of the ovary. Peritoneal dissemination and abnormal ascites were only observed in stage III–IV MT-MCT of the ovary. These are also common imaging findings for ovarian carcinomas [26], reflecting the aggressive and invasive nature of the disease. A previous study comparing CT findings of MT-MCT with BMCT of the ovary found that MT-MCTs demonstrated invasion (2/6) and dissemination (3/8) unlike BMCTs [15]. If a fat-containing ovarian mass is observed, irregular margins, peritoneal dissemination, or abnormal ascites would be reliable findings for accurately diagnosing MT-MCT of the ovary, especially in stage III–IV MT-MCT.

The solid components were more common in this study, and the maximum diameter was larger in MT-MCT than in BMCT of the ovary. A previous study examining MRI of BMCT of the ovary found that enhanced solid components are observed in 18.8%, with a mean maximum diameter of 18 mm [16]. These results are consistent with our results. Conversely, the solid components of BMCT are uncommon and small.

The common configuration of solid components in the present study was endophytic or exophytic sessile in MT-MCT and endophytic papillary in BMCT. Squamous cell carcinoma is the most prevalent histological subtype of malignant components in MT-MCT. Owing to its aggressive and invasive character, cutaneous squamous cell carcinoma MRI findings show a flattened configuration and protrusion into subcutaneous tissue [27]. On MRI, the solid components of ovarian BMCT pathologically comprised glial or thyroid tissue and formed an acute angle with the wall (71%) [16]. The sessile configuration of the solid components might be a diagnostic biomarker for MT-MCT of the ovary.

According to this study, the signal intensity ratio of solid components on T2-weighted images was lower in MT-MCT than in BMCT of the ovary and no significant difference in ADC value was observed between the two pathologies. Histopathologically, the solid components of MT-MCT comprise almost pure squamous cell carcinoma, whereas the solid components of BMCT comprise cutaneous adnexal structures and adipose tissue rather than glial or thyroid tissue [2, 10]. Squamous cell carcinoma usually shows lower signal intensity than adnexal structure and adipose tissue on T2-weighted images. Although glial or thyroid tissue in BMCT may show similar signal intensity with squamous cell carcinoma on T2-weighted images, BMCT usually have a low proportion of glial or thyroid tissue. Meanwhile, BMCT usually has a mixture of low ADC areas (keratin and fat) and high ADC areas (the other benign components); therefore, ADC value would not significantly differ between MT-MCT and BMCT.

This study has several limitations. First, the study only included a small number of patients with MT-MCT. Second, contrast-enhanced MRI was not conducted in four patients with MT-MCT. Third, we were unable to examine dynamic contrast-enhanced MRI data because only one patient with MT-MCT and ten with BMCT was performed. Finally, diffusion-weighted imaging was not performed on all the patients with MT-MCT.

In conclusion, MT-MCT exhibited a larger maximum diameter of the total tumor and solid components, lower occupancy rate of fat components, and sessile configuration of solid components. Only MT-MCT exhibited irregular tumor margins, peritoneal dissemination, and abnormal ascites. Conversely, BMCT exhibited a greater occupancy rate of fat components and papillary configuration of solid components. These MRI components allow for an accurate preoperative identification of MT-MCT vs. BMCT of the ovary.
